# The adipokine Retnla deficiency increases responsiveness to cardiac repair through adiponectin-rich bone marrow cells

**DOI:** 10.1038/s41419-021-03593-z

**Published:** 2021-03-22

**Authors:** Yong Sook Kim, Hyang Hee Cho, Dong Im Cho, Hye-yun Jeong, Soo yeon Lim, Ju Hee Jun, Mi Ra Kim, Bo Gyeong Kang, Meeyoung Cho, Hye-jin Kang, Wan Seok Kang, Goo Taeg Oh, Youngkeun Ahn

**Affiliations:** 1 Cell Regeneration Research Center, Chonnam National University Hospital, Gwangju, Republic of Korea; 2 Biomedical Research Center, Chonnam National University Hospital, Gwangju, Republic of Korea; 3 Department of Molecular Medicine, Graduate School, Chonnam National University, Gwangju, Republic of Korea; 4 Department of Life Sciences, Ewha Womans University, Seoul, Republic of Korea; 5 Department of Cardiovascular Medicine, Chonnam National University Medical School, Gwangju, Republic of Korea; 6 Department of Cardiology, Chonnam National University Hospital, Gwangju, Republic of Korea

**Keywords:** Apoptosis, Interleukins, Acute inflammation, Heart failure

## Abstract

Resistin-like alpha (Retnla) is a member of the resistin family and known to modulate fibrosis and inflammation. Here, we investigated the role of Retnla in the cardiac injury model. Myocardial infarction (MI) was induced in wild type (WT), Retnla knockout (KO), and Retnla transgenic (TG) mice. Cardiac function was assessed by echocardiography and was significantly preserved in the KO mice, while worsened in the TG group. Angiogenesis was substantially increased in the KO mice, and cardiomyocyte apoptosis was markedly suppressed in the KO mice. By Retnla treatment, the expression of p21 and the ratio of Bax to Bcl2 were increased in cardiomyocytes, while decreased in cardiac fibroblasts. Interestingly, the numbers of cardiac macrophages and unsorted bone marrow cells (UBCs) were higher in the KO mice than in the WT mice. Besides, phosphorylated histone H3(+) cells were more frequent in bone marrow of KO mice. Moreover, adiponectin in UBCs was notably higher in the KO mice compared with WT mice. In an adoptive transfer study, UBCs were isolated from KO mice to transplant to the WT infarcted heart. Cardiac function was better in the KO-UBCs transplanted group in the WT-UBCs transplanted group. Taken together, proliferative and adiponectin-rich bone marrow niche was associated with substantial cardiac recovery by suppression of cardiac apoptosis and proliferation of cardiac fibroblast.

## Introduction

Ischemic heart disease is associated with high morbidity and mortality with an enormous clinical and economic burden worldwide^1^. Cardiomyocyte loss and myocardial fibrosis are major determinants of pathological progress. It has been reported that cardiac injury leads to the activation and proliferation of cardiac fibroblasts with excessive extracellular matrix deposit that compromise myocardial structure and function^2^. Although extensive experimental findings demonstrate that endogenous regenerative factors and stem cells contribute to ventricular recovery, no current therapeutic modality for heart disease addresses the repair of lost myocardial tissue in the clinical settings^3,4^.

Resistin-like alpha (Retnla), also known as found in inflammatory zone 1 (Fizz1), is a member of cysteine-rich secreted family of Fizz/Resistin-like molecule^5,6^. Its expression has been reported in white adipose tissue and mammary gland, and is highly inducible in eosinophils and macrophages^6–9^. Retnla displayed unique functions depending on the cell types and stimuli used. Retnla stimulated α-smooth muscle actin (α-SMA) and collagen I production by fibroblasts to act as a profibrotic factor^10^. In anti-inflammatory macrophages, Retnla was well known to be induced and used as a marker of alternatively activated macrophages^11^.

In vascular disease studies, Retnla was suggested to be angiogenic, stimulating cell proliferation of rat pulmonary microvascular smooth muscle cells, and increasing angiogenesis^12^. In hyperlipidemic mice, Retnla exerted atheroprotective effect to improve the circulating lipoprotein profile via the upregulation of cholesterol-7-α-hydroxylase leading to increased excretion of cholesterol in the form of bile acids^13^. However, the relevance of Retnla to cardiac injury is currently unknown and the molecular mechanisms that regulate their specific contributions in response to cardiac injury are yet to be defined.

In this study, we observed cardiac dysfunction was attenuated in Retnla knockout (KO) mice, and we aimed to determine the beneficial mediator to further develop the therapeutic modality for cardiac repair.

## Materials and methods

### Myocardial infarction model

The animal experimental protocol was approved by the Chonnam National University Animal Care and Use Committee (CNU IACUC-H-2016-36). Retnla KO and transgenic (TG) mice for Retnla overexpression were generously provided by Dr. Goo Taeg Oh (Ewha Womans University, Seoul, Republic of Korea)^13^. Myocardial infarction (MI) was induced in 8-week-old male mice by occlusion of the coronary artery. Briefly, the mice were anesthetized with an intramuscular injection of ketamine (50 mg/kg) and xylazine (10 mg/kg), and the left anterior descending artery (LAD) was occluded within the myocardium between the left atrial appendage and the right ventricular outflow tract, using a curved needle and a 5-0 silk suture. The non MI group underwent the same surgical procedure without LAD ligation. For an adoptive transfer study, unsorted bone marrow cells (UBCs; 1 × 10^6^) were injected into infarcted myocardium right after ligation of LAD. The body weight of mice was monitored throughout the study.

### Evaluation of cardiac function

Left ventricular function was assessed by transthoracic echocardiography in a blinded manner. Two weeks after MI, the mice were anesthetized, and echocardiography was performed with a 15 MHz linear array transducer system (iE33 system, Philips Medical Systems) by an expert, who was not aware of the experimental conditions to exclude bias. To accomplish the echocardiographic analyses, interventricular septal thickness (IVS), left ventricular internal dimensions (LVID) and left ventricular posterior wall thickness (LVPW) at diastole and systole (IVSd, LVIDd, LVPWd and IVSs, LVIDs, LVPWs, respectively) are measured. End-diastolic volume, end-systolic volume, LV ejection fraction (EF), LV fractional shortening (FS), and stroke volume were also determined.

### Histology

The animals were sacrificed and the heart tissues were fixed with formaldehyde, embedded in paraffin, and sliced into 6 μm thick sections for immunohistochemical analysis. Sections were first treated with proteinase K for 15 min at 37 °C for antigen retrieval. After nonspecific binding was blocked with 5% normal goat serum (Sigma-Aldrich), the slides were incubated with primary antibodies for 18 h at 4 °C. The sections were washed three times with PBS and incubated with secondary antibodies conjugated with Alexa Fluor-594 or Alexa Fluor-488 for 1 h. After washing, the slides were mounted with a mounting medium (VectaMount mounting medium, Vector Laboratories Inc.). Blood vessel formation was detected by von Willebrand Factor (vWF) and α-SMA 14 days after MI. For assessing cardiac fibrosis, sections were stained using Trichrome Stain Kit according to the manufacturer’s protocol (ab150686, Abcam). Histological features were analyzed in the peri-infarct zone and infarct zone, and images were obtained and digitized on a computer using an Eclipse Ni microscope equipped with DS-Ri2 camera (Nikon). Antibodies used in this study were listed in Supplementary Table 1.

### Flow cytometric analysis of cardiac macrophages

Single cells were isolated from the heart tissues with as previously reported with minor modifications^7^.

Harvested hearts were minced and digested in enzyme mixture solution containing 675 U/mL collagenase I (Sigma-Aldrich), 187.5 U/mL collagenase XI (Sigma-Aldrich), 90U DNase I (Sigma-Aldrich), and 90 U/mL hyaluronidase (Worthington Laboratories) in PBS with Ca^2+^/Mg^+2^ for 60 min at 37 °C with gentle shaking. After incubation, the digestion mixture was homogenized through a 70-μm nylon mesh. Digestion mixture were centrifuged at 2000 r.p.m. for 15 min at 4 °C and the pellet was suspended 40% Percoll (GE-Healthcare) solution, than carefully flushed 80% Percoll solution under 40% Percoll solution using a pasture pipette. The tubes were centrifuged at 2200 r.p.m. for 25 min at room temperature. After Percoll gradient centrifugation, cardiac leukocytes were collected from the layer between the Percoll solutions. To get an enough cell number for flow cytometric analysis, we pooled the cells isolated three heart tissues together into a one tube. So, a sample might be considered a pool of cells from three mice. Isolated cells were stained with antibodies at 4 °C for 15 min simultaneously and were washed, resuspended in staining buffer and analyzed. Antibodies against Ly6G (127641), CD45 (103113), CD11b (101241), CD11c (101245), CD206 (141712), and F4/80 (123133) were obtained from BioLegend (San Diego, CA). The initial gating strategy evaluated the Ly6G(−) cells over CD45(+) total leukocyte population, and macrophages were identified as Ly6G(−)CD11b(+)F4/80(+) cells. The characterization of anti-inflammatory phenotype was performed using CD206, whereas inflammatory phenotype was investigated using CD11c antibodies. Cells were then washed in PBS before acquisition and analysis (BD FACSCANTO II, BD Biosciences), and data analysis was performed using FlowJo (Tree Star).

### Antibody array

Expression profiles of inflammation- and angiogenesis-related mediators in the heart tissue were detected by using the mouse antibody array membranes (ab133999, ab13967, Abcam), according to the manufacturer’s instructions. Briefly, antibody array membranes were blocked in 2 mL of blocking buffer for 1 h, and then incubated with 2 mL of the samples and antibody mixtures overnight at 4 °C. The sample mixtures were then decanted from each container, and the membranes were washed three times with 2 mL of wash buffer at room temperature with shaking. The membranes were then incubated in 1:2000 diluted streptavidin–horseradish peroxidase at room temperature for 30 min, and the membranes were washed thoroughly and exposed to a peroxidase substrate before imaging.

### Isolation of neonatal rat cardiomyocytes, cardiac fibroblasts, and adult cardiac fibroblasts

Primary cardiomyocytes and cardiac fibroblasts were isolated from 2-day-old Sprague–Dawley rats. Briefly, neonatal ventricles from neonatal rats euthanized by decapitation were separated and washed in cold PBS, chopped, and digested with 0.1% collagenase type 2 (210 U/mL, LS004176, Worthington Laboratories) and pancreatin (0.6 mg/mL, P3292, Sigma-Aldrich) for 30 min with mild stirring. The supernatant was collected for centrifugation through a Percoll (17-0891-02, GE-Healthcare) gradient at 1000 r.p.m. for 5 min. The cardiac cell layer was collected and cultured in a flask with DMEM (Invitrogen) supplemented with 10% heat-inactivated fetal bovine serum (FBS). After 1 h, the non-adherent cardiomyocyte population was removed from the adherent cardiac fibroblasts. To isolate adult cardiac fibroblasts, heart tissues were collected from mice with MI for 4 days. Tissues were rinsed, chopped, and digested with digestion buffer (100 U/mL collagenase II and 0.1% Trypsin in Hank’s balanced salt solution) for 5 min at 37 °C. Supernatant was discarded, and second digestion was followed for 20 min with gentle agitation. Supernatant was collected into a new tube with digestion buffer on ice, and further digestion was repeated at least seven times. Supernatant was harvested and pre-plated on a culture dish. After 2 h, plated cells were washed with warm PBS three times, and then further cultured on 1% gelatin-coated cell culture dishes in a DMEM/F12 supplemented with 10% FBS. Cardiomyocytes were used at passage 1 and cardiac fibroblasts were used at passages 2 and 3. Cells were treated with recombinant Retnla protein (Cat. 450-26, PeproTech Korea), AdipoRon (Cayman Chemical), doxorubicin (Doxo; #5927, Cell Signaling Technology), and glucose (G8270, Sigma-Aldrich) as indicated.

### Isolation of unsorted cells, macrophages, and mesenchymal stem cells from bone marrow

For isolation of UBCs, mice were sacrificed by cervical dislocation and the femur and tibia bones were dissected. The ends of the bones were cut and the marrow was washed out with RPMI-1640 media using a 1 mL insulin syringe (26 gauge needle), and then were transferred to a 50 mL sterile tube and sieved through a 70 μm mesh to remove debris. Red blood cell lysis buffer (BioLegend) was treated to cells followed by incubation on ice for 5 min, and then UBCs were harvested by centrifugation at 1200 r.p.m. for 10 min. The supernatant was removed and UBCs were washed with PBS, and the cell number was counted. For bone marrow-derived macrophages (BMDMs), bone marrow cells were cultured for 7 days in macrophage differentiation media (30% L929 cell-conditioned medium, 20% FBS, and 50% RPMI-1640). L929 cell-conditioned medium was prepared by growing L929 cells in RPMI-1640-containing 10% FBS for 10 days. The medium containing macrophage colony-stimulating factor secreted by the L929 cells was harvested and passed through a 0.22 mm filter. BMDMs were stimulated with LPS (100 ng/mL, L4391, Sigma-Aldrich) and IFN-γ (30 ng/mL, PHC4031, ThermoFisher Scientific) for 24 h with or without Retnla protein (500 ng/mL). Bone marrow-derived mesenchymal stem cells (MSCs) were cultured, as previously reported^14^. RAW264.7 murine macrophages cell were purchased from the Korean Cell Bank (Seoul, Korea), and cultured in DMEM media supplemented with 10% FBS, without antibiotics.

### Western blot

Cells were washed with ice-cold PBS, lysed in lysis buffer (20 mM Tris-HCl pH 7.4, 0.1 mM EDTA, 150 mM NaCl, 1 mM phenylmethylsulfonyl fluoride, and 1 mg/mL leupeptin, Sigma-Aldrich) on a rotation wheel for 1 h at 4 °C. After centrifugation at 10,000 × *g* for 10 min, the supernatant was prepared as a protein extract. Equal concentrations of proteins were fractionated by electrophoresis on acrylamide gels and were transferred onto a polyvinylidene fluoride membrane (IPVH00010, Millipore) membrane, followed by blotting with antibodies against followed by secondary staining with horseradish peroxidase-conjugated IgG. Proteins expression was detected using Image Reader (LAS-3000 Imaging System, Fuji Photo Film). The expression level was quantified by ImageJ (NIH). Antibodies used in this study are described in Supplementary Table 1.

### Real-time PCR

In all cases, total RNA from BMDM cocultured with MSC were extracted with Trizol® Reagent (Invitrogen) and chloroform, and RNA samples were converted to cDNA using an Applied Biosystems® High-Capacity cDNA Reverse transcription Kit (Invitrogen), according to the manufacturer’s instructions. Real-time PCR was performed using a QuantiTect SYBR Green PCR kit (QIAGEN) and Corbett Research Rotor-Gene RG-3000 Real-Time PCR System. All results were processed with 2^−ΔΔCT^ method after normalizing to GAPDH. Primers used in PCR are described in Supplementaary Table 2.

### Enzyme-linked immunosorbent assay

The protein levels of adiponectin (MRP300, R&D systems), interleukin-6 (IL-6, BMS603-2, Invitrogen), IL-1β (BMS6002, Invitrogen), IL-18 (BMS618-3, Invitrogen), and Retnla (MBS9394009, MyBioSource) in mouse plasma were evaluated by using enzyme-linked immunosorbent assay (ELISA) kits. Inflammatory mediators in mouse plasma were evaluated by Multi-Analyte ELISArray kit (Qiagen, 336161).

### Immunofluorescence staining of cardiomyocytes and cardiac fibroblasts

To assess the expression of adiponectin (APN) in cardiomyocytes, cells were fixed with 4% paraformaldehyde or ice-cold methanol for 10 min and washed with PBS three times. After permeabilization by 0.1% Triton X-100 for 10 min and blocking with 5% BSA for 1 h, primary antibodies for adiponectin and cardiac troponin I (cTnI) were incubated overnight at 4 °C, followed by sequential incubation with secondary antibody conjugated with Alexa-fluor-488 or Alexa-fluor-594 (Cell Signaling Technology). The proliferation of cardiac fibroblasts was measured by staining with phosphorylated histone H3 (pH3) or Ki67. Cell proliferation was induced by 3% FBS treatment with or without Retnla treatment (500 ng/mL) for 24 h. Proliferating cells which were positive to pH3 or Ki67 were counted and quantified. After washing with PBS, the slides were mounted with medium containing DAPI (Invitrogen) and observed under fluorescence microscopy.

### In vitro angiogenesis assay

Tube formation was assayed by using an in vitro angiogenesis assay kit (Merck Millipore). Cells were plated onto matrix gel coated 96-well plates and cultured in DMEM without serum. Tube formation was monitored and photographed, and images were analyzed by using Image-Pro software. Angiogenic activity was quantified by measuring tube length, area, and branching points.

### Microfil casting

Mice were sacrificed at 14 days of MI to observe the vasculature. Mice were euthanized by CO_2_ inhalation, and the thoracic cavity was opened surgically. The large branches from the aorta were ligated. The vasculature was flushed with normal saline containing heparin (200 U/mL) via a needle inserted into the descending aorta until heart became visibly blanched. The heart was then pressure-fixed with 2% paraformaldehyde. Paraformaldehyde was flushed from the heart in heparinized saline, and coronary vasculature was injected with Microfil (MV-122; Flowtech) solution prepared in a volume ratio of 1:1 of Microfil diluent with 5% curing agent. Once filling is complete, to prevent the Microfil leakage from the coronary vessels, the accessible major vascular exit points were ligated immediately after filling. Heart was stored at 4 °C for contrast agent polymerization.

### Statistical analysis

Experimental results were expressed as the mean ± standard error of the mean. The number of experiments or animal is indicated. Differences between groups were tested by one-way analysis of variance, and comparisons between two groups were evaluated using the Student *t* test. Datasets of western blot, real-time PCR, and ELISA were presented in column bar graph format to show the fold changes, and others were presented in scatter plot with bar format to show the distribution of the data points. GraphPad Prism (version 5.00, La Jolla, USA) was used for all analysis and *P* < 0.05 was considered statistically significant.

## Results

### Retnla-deficient mice exhibit the amelioration in cardiac dysfunction, inflammation, and angiogenesis as compared to wild type in response to MI

**Fig. 1:**
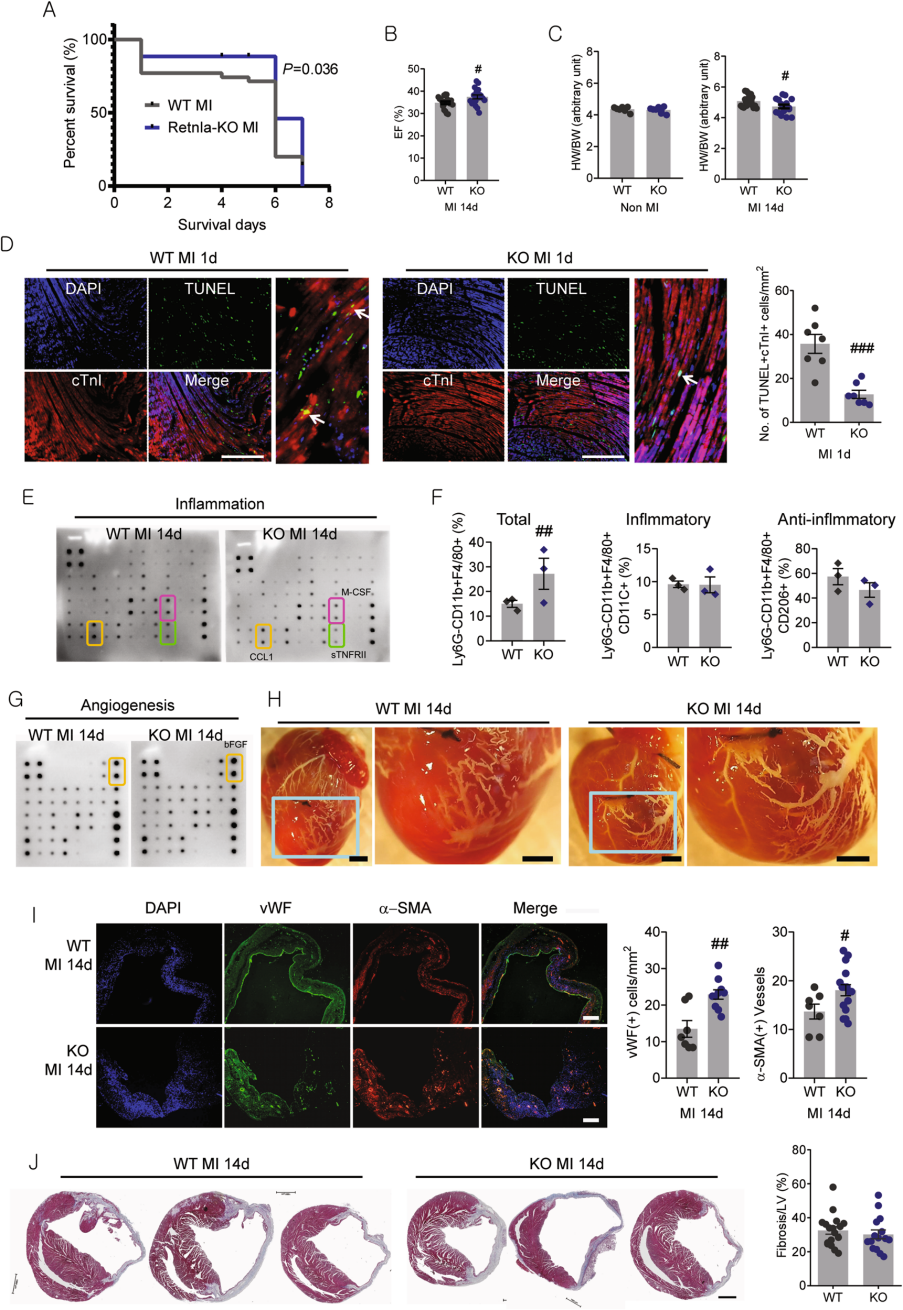
Retnla knockout protects mouse heart from myocardial infarction injury. **A** Survival rates of the experimental groups; wild type (WT, *n* = 15) mice and Retnla knockout (KO, *n* = 17) mice with or without myocardial infarction (MI). WT MI *n* = 47, KO MI *n* = 36. **B** Left ventricular ejection fraction (LVEF) was determined by echocardiography in Retnla KO (*n* = 19) and WT littermates (*n* = 18) 14 days after MI. **C** Heart weight/body weight (HW/BW) ratios of mice were calculated before and at 14 days after MI. (WT; *n* = 7, KO; n = 7, WT MI; *n* = 18, KO MI; *n* = 16). **D** Cardiomyocyte apoptosis (white arrows) in the peri-infarct area was determined by TUNEL staining. cTnI cardiac troponin I. Scale bar, 200 μm. (WT; *n* = 7, KO; *n* = 7). **E** Inflammation-related mediators were analyzed by using antibody array membranes. **F** Three days after MI, cardiac macrophages were isolated to compare the cell number and phenotype. Ly6G(−)CD11b(+)F4/80(+)CD11C(−) cells are pro-inflammatory macrophages and Ly6G(−)CD11b(+)F4/80(+)CD206(+) cells are anti-inflammatory macrophages. The number of Ly6G(−)CD11b9(+)F4/80(+) cardiac macrophages was higher in the KO mice (*n* = 3, three mice per sample each) than in the WT mice (*n* = 3, three mice per sample each). **G** Angiogenesis-related mediators were analyzed by using antibody array membranes. **H** Microfil perfusion of hearts from WT and Retnla KO mice with MI for 14 days. **I** Left ventricular vascularity was assessed by immunostaining of von Willebrand factor (vWF) and α-smooth muscle actin (α-SMA). Representative images of vWF stained capillaries and α-SMA(+)-vessels in the infarcted heart. (WT; *n* = 7, KO; *n* = 10). Scale bar, 100 μm. **J** Representative Masson’s trichrome staining images of WT (*n* = 17) and Retnla KO mice (*n* = 15) after 14 days of MI. Quantitative analysis of ventricular fibrosis (expressed as a percentage of total left ventricular area) in WT and KO. Scale bar, 1000 μm. EF ejection fraction, FS fractional shortening. Data are represented as mean ± SEM. #*P* < 0.05, ##*P* < 0.01, ###*P* < 0.001 (by Student’s *t* test).

The survival rate after MI was higher in the Retnla KO mice than in the wild-type (WT) mice (*P* = 0.036, Fig. 1A). Then experimental MI-induced cardiac function was assessed in the Retnla KO mice and the WT mice. Echocardiographic assessments showed that without MI, WT, and Retnla KO mice had similar cardiac dimensions and function (Fig. S1A). However, LVEF was considerably higher at 14 days in the Retnla KO mice than in the WT mice (Fig. 1B and Fig. S1B, C). The increases in heart weight-to-body-weight ratio 14 days after MI was significantly attenuated in Retnla KO mice compared with WT mice (Fig. 1C).

To compare the cardiomyocyte loss, cardiac apoptosis was examined by TUNEL staining after 24 h of MI. CTnI(+)TUNEL(+) cells were identified as apoptotic cardiomyocytes, and the number of cardiac apoptosis was lower in the KO mice than in the WT mice (Fig. 1D). Although cardiac remodeling indexes, such as LVIDd or LVIDs, did not show significant changes (Fig. S1B), remarkable reduction of cardiomyocyte apoptosis could be contributable to the improvement of cardiac function in the KO group (Fig. 1D).

Then, we compared the levels of inflammation and angiogenesis-related mediators from the heart tissues by using antibody array. The inflammation-related CCL1 (C–C motif chemokine ligand 1), M-CSF (colony-stimulating factor 1), and soluble TNFRII (TNF receptor superfamily member 1b) were lower in the KO mice than in the WT mice (Fig. 1E). CCL1 is a potent chemoattractant for monocytes and macrophages, and is involved in stimulating pathological inflammation in heart failure patients^15^. M-CSF is a causal inflammatory biomarker in coronary artery disease and a strong predictor of atherosclerotic plaque progression and adverse outcome^16,17^. Circulating soluble TNFRII was elevated in patients with heart failure^18^ and showed high correlation with severity of heart failure^19,20^.

The phenotype of macrophages infiltrated into infarcted heart tissue is associated with pathological progress. At day 3, cardiac macrophages were isolated to analyze the phenotypes. The numbers of Ly6G(−)CD11b(+)F4/80(+)CD11c(+) pro-inflammatory macrophages and Ly6G(−)CD11b(+)F4/80(+)CD206(+) anti-inflammatory macrophages were not different in the both groups. Unexpectedly, flow cytometry analysis showed that the number of Ly6G(−)CD11b(+)F4/80(+) cardiac macrophages of KO mice nearly twice that of WT mice (Fig. 1F).

Angiogenesis-related antibody array showed that basic fibroblast growth factor was higher in the KO mice than in the WT mice (Fig. 1G), indicating higher angiogenic capacity. To better visualize the collaterals, we perfused the vasculature with a low viscosity compound (Microfil, Flowtech) and Retnla KO mice displayed better developed collaterals (Fig. 1H), implicating the improvement of angiogenesis in the infarcted myocardium. Then, angiogenesis was assessed and we found that Retnla KO mice have increased numbers of vWF(+) cells and α-SMA(+) cells in the infarcted myocardium by 14 days post MI (Fig. 1I). In non MI mice, vWF(+) cells were rarely observed, and double IHC images were not different between the WT and the KO group (Fig. S1C). Together, these results implicated reduced inflammation and increased angiogenic response in Retnla KO mice.

The cardiac fibrosis was not significantly different; 32.57 ± 9.51% in the WT group and 30.20 ± 10.22% in the KO group 14 days after MI (Fig. 1J). Moreover, the fibrotic changes were 12.27 ± 2.69% and 19.16 ± 6.32% in the WT group, and 11.06 ± 3.09% and 18.20 ± 1.08% in the KO group at day 4 and 7, respectively (Fig. S2A, B).

These results indicated that Retnla deficiency exerted cardioprotective effect mainly mediated by suppression of cardiac apoptosis, not by modulations of macrophage phenotype and fibrotic progress.

### High proliferation rate, adiponectin level, and angiogenic activity of Retnla-deficient unsorted bone marrow cells are associated with improvement of cardiac function following MI

**Fig. 2:**
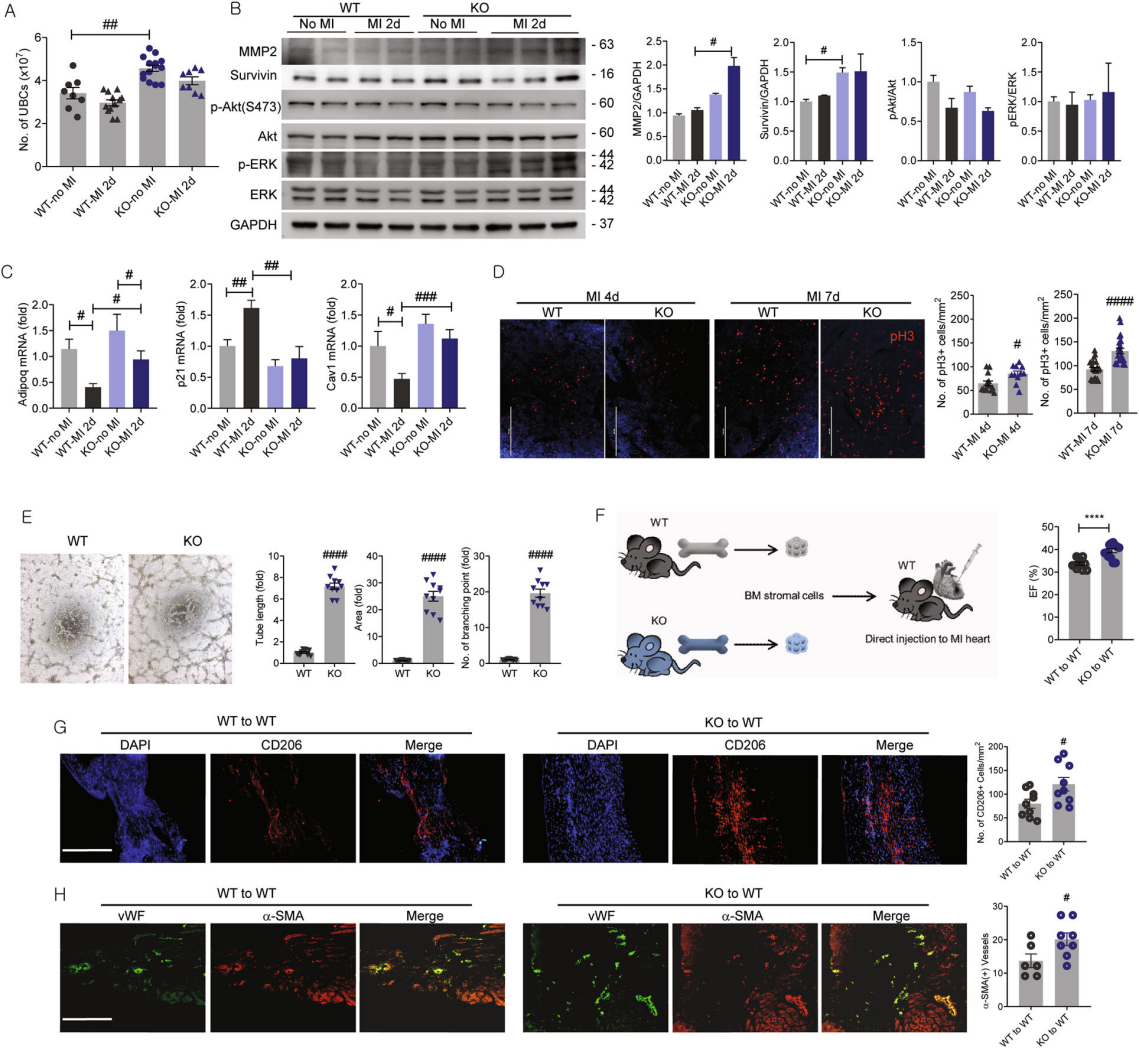
Characterization of Retnla knockout in unsorted bone marrow cells exhibiting cardiac protective effect. **A** The number of unsorted bone marrow cells (UBCs) was higher in Retnla knockout (KO) mice regardless of myocardial infarction (MI). **B** Cell survival and proliferation-related mediators were analyzed in UBCs isolated from WT and Retnla KO mice by western blot. **C** UBCs were isolated from WT and KO mice 2 days after MI, and mRNA levels of adiponectin (Adipoq), p21, and caveolin-1 (Cav1) were quantified by real-time PCR. **D** The number of phosphorylated histone H3 (pH3)-positive cells in bone marrow was quantified, and the representative images showed pH3-positive cells (red fluorescence) in the bone marrow. (WT MI 4d; *n* = 14, KO MI 4d; *n* = 11, WT MI 7d; *n* = 23, KO MI 7d; *n* = 23). Scale bar, 200 μm. **E** To compare the endogenous angiogenic potential of WT and KO mice, mesenchymal stem cells (MSCs) were used. MSCs were isolated from bone marrow and tube formation assay was performed. Tube length, tubular area, and the number of branching points were analyzed in MSCs from wild type and Retnla KO mice. Scale bar, 500 μm. **F** UBCs were isolated from WT (*n* = 12) or KO mice (*n* = 13), and injected into infarcted heart of wild-type mice (*n* = 12 in the WT to WT mice, *n* = 13 in the KO to WT mice). After 2 weeks, cardiac function was analyzed, and the representative echocardiograms were shown. **G**, **H** Immunofluorescence staining showed larger distributions of anti-inflammatory CD206(+) macrophages and vascular density in the KO mice than in the WT mice. EF ejection fraction, FS fractional shortening. Scale bars, 200 μm. Data are represented as mean ± SEM. #*P* < 0.05, ##*P* < 0.01, ###*P* < 0.001 (by Student’s *t* test or two-way ANOVA). The image of the mouse (http://clipart-library.com/clipart/724544.htm) is obtained from clipart library and used under the Creative Commons Attribution-Share Alike 4.0 International license (http://creativecommons.org/licenses/by-sa/4.0/).

As shown in Fig. 1F, the total number of cardiac macrophages was higher in Retnla KO mice. Bone marrow-derived circulating monocytes are known to infiltrate into the damaged heart tissue and then differentiate to macrophages^21^. To find out whether left ventricular dysfunction post-MI was relevant to bone marrow niche, we isolated UBCs from bone marrow and found the number of UBCs was higher in the Retnla KO mice than in the WT mice regardless of MI (Fig. 2A). To explore the proliferative mediators, UBCs were isolated from bone marrow of non MI and 2 days post MI mice to analyze proliferation-related factors by western blotting. Protein levels of MMP2 in the KO-derived UBCs were higher in the MI groups. Basal level of survivin was higher in KO-derived UBCs, indicating higher proliferative capacity. However, phosphorylated Akt (Ser473) and extracellular signal-regulated kinase (ERK) did not show significant differences in UBCs (Fig. 2B). Retnla is a member of resistin family, and adiponectin is the representative adipokine with opposite effects of resistin. Adiponectin is known to be cardioprotective, so we determined the adiponectin expression to define its involvement and adiponectin expression was higher in KO-UBCs than in WT-UBCs both at baseline and 2 days of MI. Besides, p21 and caveolin-1 (Cav1) stayed unchanged even after MI in the KO mice not in the WT mice (Fig. 2C). Cav1 was found to promote or suppress cell proliferation dependent on the cell types by regulating apoptosis^22,23^. Unlikely in WT-UBCs, p21 did not elevated while Cav1 did not downregulated in KO-UBCs by MI. Based on these results, we approached to compare the proliferating cells in the bone marrow niche by detecting proliferative cells. pH3 is a sensitive and specific marker for mitosis^24^, and the number of pH3(+) cells was significantly higher in the KO bone marrow than in the WT bone marrow after MI 4 days and 7 days (Fig. 2D). Next, to assess whether the endogenous potential of angiogenesis was different, MSCs were isolated from bone marrow and tube formation was assessed by in vitro angiogenesis assay. Tube length, tube area, and tube sprouting were substantially higher in the KO mice than in the WT mice (Fig. 2E). Furthermore, an aortic ring assay also showed sprouting microvessels were much more abundant in KO group than in the WT group (Fig. S3). These results suggested that Retnla KO mice have highly proliferative, angiogenic, and adiponectin-rich bone marrow niche.

Next, we performed an adoptive transfer study to investigate whether UBCs of Retnla KO mice recapitulate the benefits in progress after MI. UBCs were isolated from Retnla KO mice and WT mice, and then injected into infarcted myocardium of WT mice. Cardiac function of recipient mice was assessed at 2 weeks, and mice transplanted with KO-UBCs showed improved LVEF and LVFS after MI (Fig. 2F and Fig. S4A, B). The number of CD206(+) cells in the infarcted heart was significantly higher in the KO-derived UBCs transplantation group than in the WT-derived UBCs group (Fig. 2G). The vasculogenesis were assessed by expressions of vWF and α-SMA, and quantitative analysis showed blood vessel density was higher in the KO-UBCs transplanted group than in the WT-UBCs transplanted group (Fig. 2H). These results show that administration of KO-UBCs may have contributed to alleviate cardiac injury.

### Retnla induces cardiomyocyte apoptosis through increase the ratio of apoptotic Bax to antiapoptotic Bcl2

**Fig. 3:**
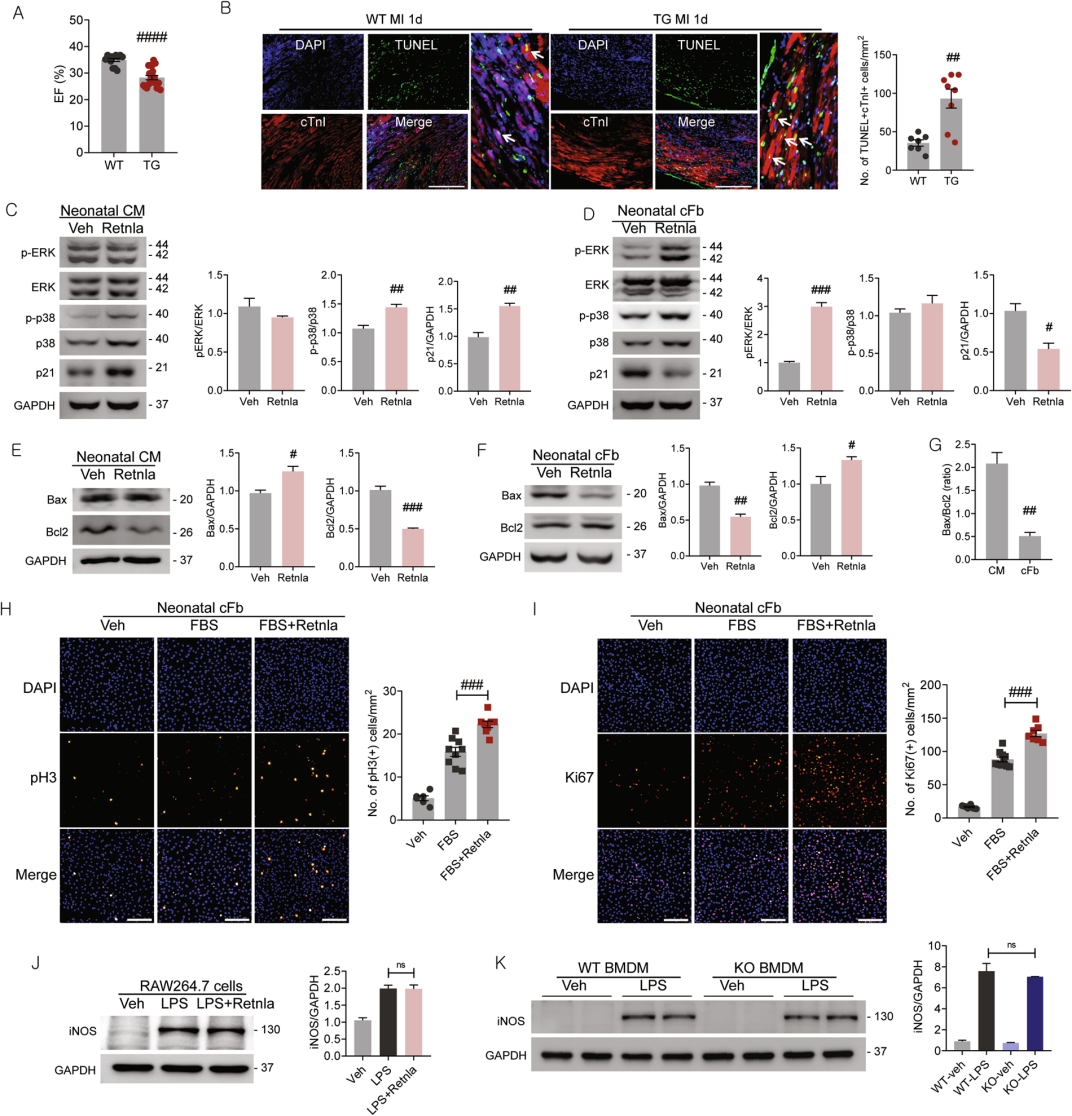
Effects of Retnla protein treatment on cardiomyocytes, cardiac fibroblasts, and macrophages. **A** Cardiac function was assessed by echocardiography in wild type (WT, *n* = 13) and Retnla transgenic (TG) mice (*n* = 20) after myocardial infarction (MI). **B** Cardiomyocyte apoptosis (white arrows) in the peri-infarct area was determined by TUNEL staining. cTnI cardiac troponin I. (WT; *n* = 7, KO; *n* = 8). Scale bar, 200 μm. **C**, **D** Phosphorylation of stress-activated ERK and p38 and cell cycle arrest-related proteins p21, and were assessed in neonatal rat cardiomyocytes (CM) or neonatal rat cardiac fibroblasts (cFb) by western blotting. Cells were treated with veh or Retnla (500 ng/mL) for 24 h. **E**, **F** Expression of pro-apoptotic Bax and antiapoptotic Bcl2 was determined by western blot analysis in cardiomyocytes and cardiac fibroblasts treated with vehicle (veh) or Retnla (500 ng/mL) protein for 24 h. **G** The ratios of Bax to Bcl2 were calculated in cardiomyocytes and cardiac fibroblasts and showed significant difference. **H**, **I** Proliferation of cardiac fibroblasts were induced with FBS with or without Retnla treatment for 24 h. Proliferating cell markers, phosphorylated histone H3 (pH3) and Ki67, were used and quantified by counting pH3(+) cells (veh; *n* = 6, FBS; *n* = 9, FBS + Retnla; *n* = 8) and Ki67(+) cells (veh; *n* = 6, FBS; *n* = 10, FBS + Retnla; *n* = 7). **J** Induction of inducible nitric oxide synthase (iNOS), a pro-inflammatory marker, was assessed in RAW264.7 cells stimulated with lipopolysaccharide (LPS, 100 μg/mL) for 24 h in the presence of Retnla (500 ng/mL). **K** Bone marrow-derived macrophages (BMDMs) were derived from WT mice and Retnla KO, and stimulated with LPS (100 μg/mL) for 24 h. Induction of iNOS was compared by western blot. Data are represented as mean ± SEM. #*P* < 0.05, ##*P* < 0.01, ###*P* < 0.001 (by Student’s *t* test or one-way ANOVA with Bonferroni’s multiple comparisons test).

Next, we compared cardiac performance between WT and Retnla TG mice. The LVEF was lower in the TG group compared with the WT mice (Fig. 3A and Fig. S5A), and TG mice showed higher number of cardiomyocyte apoptosis than WT mice (Fig. 3B).

Unexpectedly, however, there was an apparent physical characteristic with lower body weight and spleen weight in TG mice. The heart weight was not different in TG mice with other groups (Fig. S5B, C). The circulating Retnla levels were 7.62 ± 0.08 μg/mL in TG mice and 0.66 ± 0.24 μg/mL in WT mice (Fig. S5D). Based on this critical observation, we decided to rule out TG mice in this study because of the underlying unknown physiological differences between WT and TG mice. Instead of using TG mice, we treated recombinant Retnla protein to cardiomyocytes or cardiac fibroblasts to recapitulate pathophysiological condition of TG mice. To examine the effect of Retnla on proliferation or apoptosis of cardiac cells, cardiomyocytes and cardiac fibroblasts were isolated from neonatal rats and treated with Retnla protein (500 ng/mL) for 24 h.

The activation of mitogen-activated protein kinases (MAPKs), such as ERK and p38, was assessed by detection of phosphorylated MAPKs. In cardiomyocytes, phosphorylation of p38 was increased by Retnla treatment (Fig. 3C). On the other hand, Retnla-treated cardiac fibroblasts showed substantially increase in phosphorylations of ERK and p38 (Fig. 3D). p21, one of the critical cell cycle inhibitor, was upregulated in cardiomyocytes, and markedly reduced in cardiac fibroblasts by Retnla treatment (Fig. 3C, D). These data implicated proliferative activity of cardiac fibroblasts was potentiated by Retnla treatment. To assess effect of Retnla on cell apoptosis, the ratio of pro-apoptotic Bax to antiapoptotic Bcl-2 was measured. These two proteins are good indicators for cardiomyocyte apoptosis. Treatment of cardiomyocytes with apoptosis inducers, such as Doxo 1 μM or high glucose 30 mM showed typical increase of Bax and Bcl-2 decrease (Fig. S5E). The remarkable increase of Bax and decrease of Bcl-2 were remarkable in Retnla-treated cardiomyocytes (Fig. 3E). On the other hand, decrease of Bax and increase of Bcl-2 were prominent in Retnla-treated cardiac fibroblasts (Fig. 3F). Particularly, cardiac fibroblast resistance to apoptosis was known to largely contribute to pathological fibrosis^25^. Altogether, ratio of Bax to Bcl-2 was increased in Retnla-treated cardiomyocytes (2.06 ± 0.37-fold, *P* < 0.05), whereas was decreased in Retnla-treated cardiac fibroblasts (0.55 ± 0.16-fold, *P* < 0.05, Fig. 3G).

To further support the proliferative effect of Retnla on cardiac fibroblasts, we additively quantified the proliferating cells that were positive to pH3 or Ki67. FBS was used to initiate and induce cell proliferation, and Retnla treatment clearly increased the number of pH3(+) cells and Ki67(+) cardiac fibroblasts (Fig. 3H, I). These results implicated that cardiomyocytes apoptosis and cardiac fibroblast proliferation were induced by Retnla stimulation.

In macrophages, Retnla is known as a marker of anti-inflammatory phenotype, but Retnla treatment to RAW264.7 macrophages did not affect the phenotype. Inducible nitric oxide synthase (iNOS) is a marker of inflammatory macrophages, and Retnla treatment did not alter LPS-induced iNOS expression (Fig. 3J). Moreover, BMDMs were isolated from WT and KO mice, and the induction of iNOS in response to LPS treatment was not different (Fig. 3K). These results indicated that Retnla stimulated cardiomyocyte apoptosis and cardiac fibroblast activation, but not macrophage polarization.

### Beneficial effects of Retnla deficiency are associated with downregulation of mediators in apoptosis, fibrosis, and inflammation

**Fig. 4:**
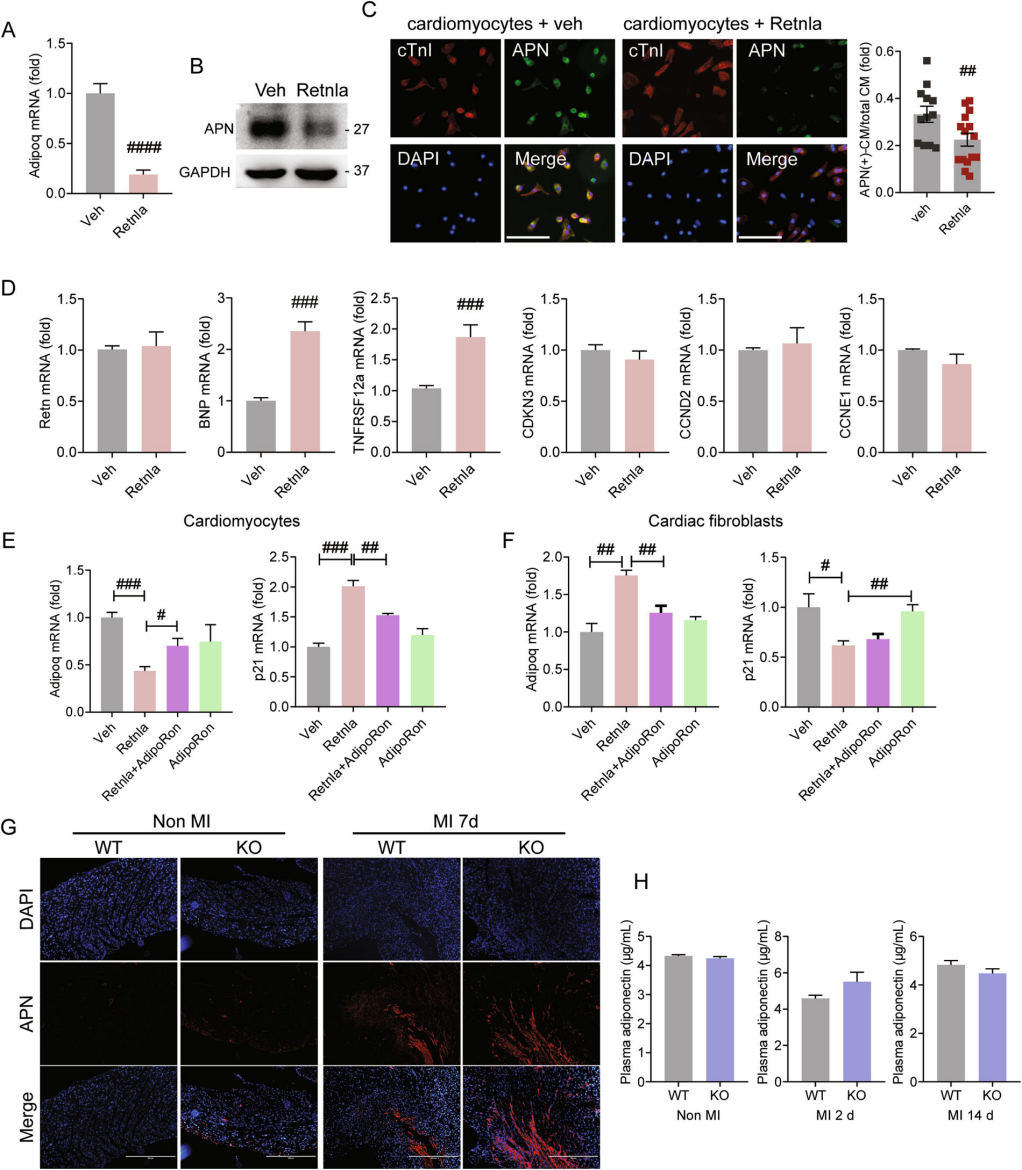
Involvement of adiponectin in cardiac protection mediated by unsorted bone marrow cells. **A** The mRNA level of adiponectin (Adipoq) was assessed in neonatal rat cardiomyocytes treated with vehicle (veh) or Retnla (500 ng/mL) for 24 h. **B** Adiponectin (APN) protein expression was determined by western blot in neonatal rat cardiomyocytes. **C** Endogenous APN expression was assessed by immunofluorescence staining in neonatal rat cardiomyocytes treated with veh (*n* = 12) or Retnla (500 ng/mL, *n* = 15). Adiponectin-expressing cardiomyocytes were counted and quantified. Scale bars, 200 μm. **D** Relative gene expression analysis of resistin (Retn), brain natriuretic peptide (BNP), tumor necrosis factor receptor superfamily member 12a (TNFRSF12A), cyclin-dependent kinase inhibitor 3 (CDKN3), cyclin D2 (CCND2), and cyclin E1 (CCNE1) in cardiomyocytes treated with veh or Retnla (500 ng/mL) for 24 h. **E**, **F** AdipoRon, adiponectin receptor agonist that binds adiponectin receptor 1 and 2, partially restored the transcriptional levels of adiponectin and p21 in Retnla-treated cardiomyocytes and cardiac fibroblasts. **G** APN protein expression was determined in the myocardium. Scale bar, 400 μm. **H** Circulating level of APN was measured in WT mice (*n* = 8) and KO mice (*n* = 8) at 0 day, 2 days, and 14 days after MI. Data are represented as mean ± SEM. #*P* < 0.05, ##*P* < 0.01, ###*P* < 0.001 (by Student’s *t* test or one-way ANOVA with Bonferroni’s multiple comparisons test).

Retnla is a member of resistin family^5^, and the effect of Retnla treatment on expressions of resistin and adiponectin were examined in cardiac cells. Adiponectin mRNA level was reduced in cardiomyocytes (Fig. 4A) by Retnla treatment. On the other hand, mRNA levels of adiponectin and resistin were markedly induced in Retnla-treated cardiac fibroblasts (Fig. S6A).

**Fig. 5:**
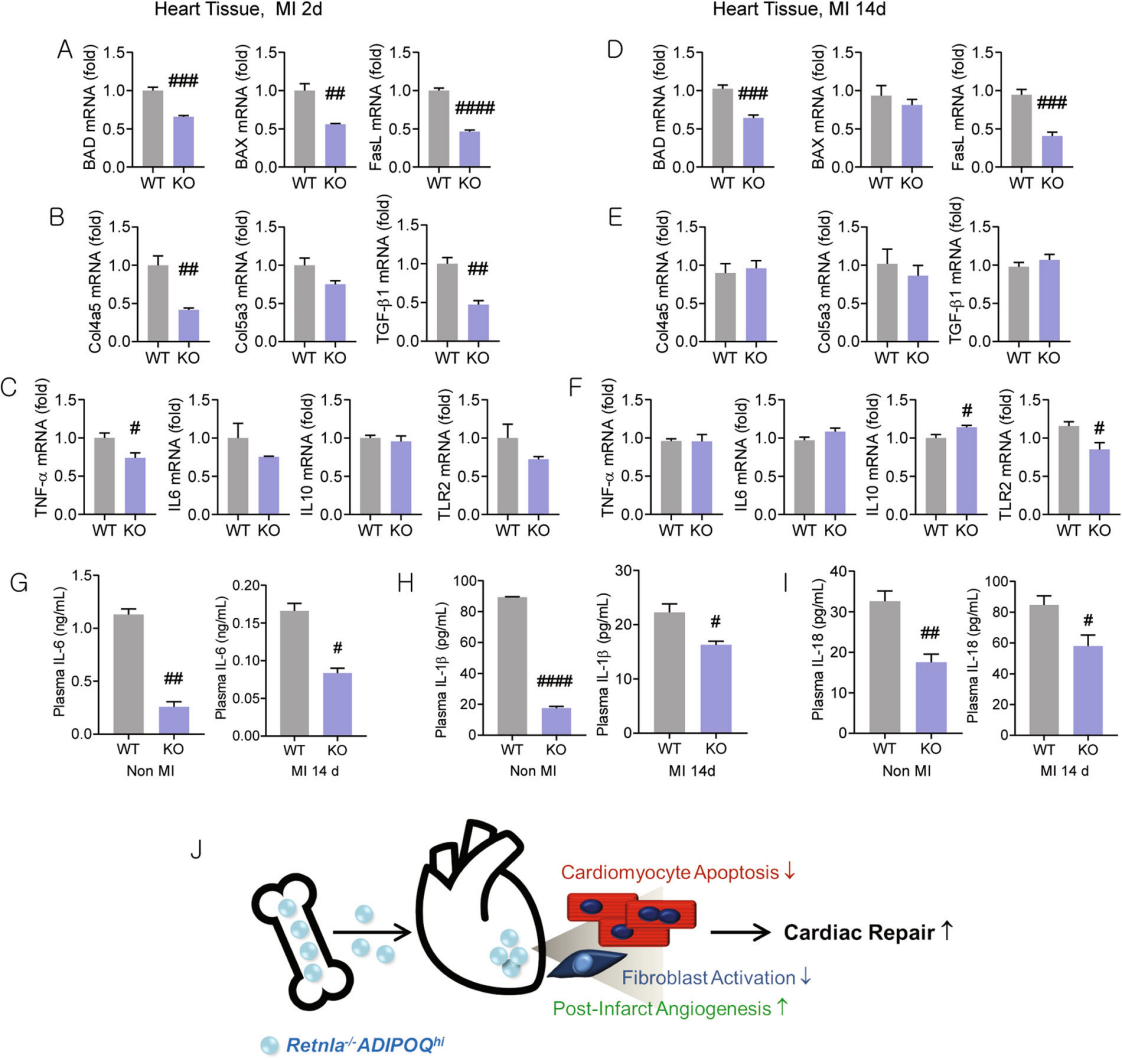
Expressional changes of signaling molecules of apoptosis, inflammation, fibrosis, and tissue repair in the heart tissues. Heart tissues were collected at day 2 (**A**–**C**) and 14 (**D**–**F**) after MI to analyzed signaling molecules. BCL2-associated agonist of cell death (BAD), BCL2-associated X, apoptosis regulator (BAX), and Fas ligand (FasL) are cell death-related molecules. Collagen type IV, α5 (Col4α5), collagen type V, α3 (Col5α3), and transforming growth factor-β1 (TGF-β1) are fibrosis-related molecules. Tumor necrosis factor-α (TNF-α), interleukin-6 (IL-6), IL-10, and toll-like receptor 2 (TLR2) are inflammation-related molecules. **G**–**I** Circulating levels of interleukin-6 (IL-6), IL-1β, and IL-18 in WT mice (*n* = 5) and KO mice (*n* = 5) with or without MI were measured. Data are represented as mean ± SEM. #*P* < 0.05, ##*P* < 0.01, ###*P* < 0.001 (by Student’s *t* test). **J** Schematic representa*t*ion showing the proliferative bone marrow milieu with high adiponectin is associated with improved cardiac repair in infarcted myocardium. The images of bone (https://www.onlinewebfonts.com/icon/559079) and heart (https://www.onlinewebfonts.com/icon/493852) is obtained from clipart library, and used under the Creative Commons Attribution-Share Alike 4.0 International license (http://creativecommons.org/licenses/by-sa/4.0/).

To assess the protein expression of adiponectin in cardiomyocytes, western blot and immunofluorescence staining were performed. Adiponectin expression in cardiomyocytes was significantly suppressed by Retnla treatment (Fig. 4B). Immunofluorescence staining also showed the number of adiponectin(+) cardiomyocytes was lower in the Retnla-treated cardiomyocytes than in the vehicle-treated cells (Fig. 4C). In cardiomyocytes, Retnla treatment upregulated cardiac injury-related molecules, such as brain natriuretic peptide and tumor necrosis factor receptor superfamily member 12a. On the other hand, resistin and cell cycle regulators, such as cyclin-dependent kinase inhibitor 3, cyclin D2, and cyclin E1 were not changed (Fig. 4D). From these results, cardiac stress was induced along with a significant downregulation of adiponectin in Retnla-stimulated cardiomyocytes. To confirm the involvement of adiponectin in Retnla-induced cardiac stress, AdipoRon, an agonist of adiponectin receptor 1 and 2, was treated in cardiomyocytes and cardiac fibroblasts. By co-treatment of AdipoRon with Retnla, the levels of adiponectin and p21 were partially restored in both cardiomyocytes and cardiac fibroblasts (Figs. 4E, F and Fig. S6B). Then, we compared adiponectin expression by immunohistochemical staining in the infarcted myocardium. Adiponectin protein, undetectable in non-infarcted heart tissue, was highly upregulated in the Retnla KO mice compared with the WT mice 7 days after MI (Fig. 4G). However, circulating adiponectin levels did not show the statistical differences between WT mice and KO mice, but showed a tendency to increase in KO mice 2 days after MI induction without statistical significance (Fig. 4H). Furthermore, real-time PCR analysis of heart tissues isolated from WT and KO mice showed a significant decrease in apoptosis markers (BAD, BAX, and FasL, Fig. 5A, D), profibrotic markers (Col4α5 and TGF-β1, Fig. 5B, E), and inflammatory mediators (TNF-α and TLR2, Fig. 5C, F) in the KO mice compared to the WT mice. Then, circulating inflammatory cytokines were measured. Interestingly, the levels of IL-6, IL-1β, and IL-18 were lower in the Retnla KO mice with or without MI (Fig. 5G–I). These data indicate that integrated and complementary mechanisms, resulting in cell death and inflammation lead to an attenuated cardiac dysfunction in the Retnla KO mice.

## Discussion

MI causes a sterile and systemic inflammatory response^26,27^. Cardiomyocyte death triggers activation of macrophages and cardiac fibroblasts proliferation^28^, and infarct maturation is associated with resolution of inflammation and scar formation^1,21,29^. The skewed macrophage polarization toward pro-inflammatory phenotype was related with contractile dysfunction^30,31^. Retnla is a well-known marker of alternatively activated anti-inflammatory macrophage^6,10^. At the beginning of this study, we envisaged Retnla KO macrophage would be defective in the timely polarization leading to the detrimental roles in post-infarct pathogenesis. Unexpectedly however, polarization of macrophages isolated from bone marrow and infarcted heart tissue was not different between WT and KO mice. Interestingly, we accidently found that bone marrow cells and peritoneal macrophages were more abundant in KO mice than in WT mice. Based on these observations, we decided to rule out macrophage polarization as a possible contributing mechanism in this study.

Retnla was originally found in lung allergic inflammation, and was known to be expressed predominantly in white adipose tissue^6^. The pathophysiological roles of Retnla are cell type specific. Retnla was markedly induced in bleomycin-induced pulmonary fibrosis and pulmonary hypertension models. Lung myofibroblast differentiation was promoted by Retnla via induction of α-SMA, whereas adipocyte differentiation and maturation were inhibited by Retnla^10,32^. In bleomycin-induced dermal fibrosis model, Retnla KO mice showed that fibrosis was significantly reduced with preservation of the subcutaneous fat and Retnla was suggested as an important regulator in pathogenesis of fibrosis^33^. Currently, there is no report about the role of Retnla in cardiac injury.

MI models in Retnla KO mice and TG mice showed contradictory results; TG mice overexpressing Retnla exhibited worsened cardiac function and cardiomyocyte apoptosis. More surprisingly, growth rate and general conditions were poor in TG mice as compared to WT littermates on chow diet. These observations implicated the significant correlation between high level of Retnla and pathological progress, and we decided to recapitulate the Retnla-overexpressing TG setting in vitro instead of using TG mice. In this study, we treated cardiomyocytes, cardiac fibroblasts, and macrophages with Retnla recombinant protein.

In good correspondence with post-MI studies in KO and TG mice, we found that Retnla treatment upregulated the ratio of Bax to Bcl2 and p21 in cardiomyocytes, which might be responsible for cardiac apoptosis in TG mice. In Retnla-treated cardiac fibroblasts, the decreases in the ratio of Bax to Bcl2 and p21, and increased phosphorylations of ERK and p38 were observed. The data presented here indicated that Retnla was involved in the anti-apoptosis and proliferation of cardiac fibroblasts to result in cardiac fibrosis. Previous studies also showed that Retnla induced myofibroblast differentiation and Retnla treatment inhibited tumor necrosis factor-α-induced activities of caspase-3 and caspase-8, indicating that Retnla might contribute to the pathogenesis of pulmonary fibrosis by induction of fibroblast resistance to apoptosis^10,34^.

However, we did not directly compare the cellular characteristics between Retnla-overexpressing cells and Retnla-deficient cells. Instead, we examined the pathophysiological roles of overexpressing Retnla on cardiomyocytes, fibroblasts, and macrophages. So, further investigations are needed to understand the cardioprotective mechanisms in the Retnla KO mice rather than interpretation of Retnla treatment study. In addition to fibroblast proliferation, cardiac fibrosis is modulated by several factors, including inflammation, signaling molecules secreted from dead cardiomyocyte, and angiogenic potential.

The levels of IL-6, IL-1β, and IL-18 in plasma were remarkably lower in the Retnla KO mice than in the WT mice with or without MI (Fig. 5G–I). Retnla KO mice exhibited that cardiomyocyte apoptosis and systemic inflammation were apparently decreased, and these could attenuate the pathological progress after MI.

Although Retnla is an anti-inflammatory marker of macrophages, both Retnla treatment and Retnla KO had no effects on polarization of macrophages. In addition, cardiac macrophages did not show the phenotypic differences between WT mice and KO mice. Retnla is a member of resistin family. Resistin is a peptide with biological properties opposite to adiponectin^35^. Due to its pro-inflammatory activity, resistin was linked to the development of insulin resistance and type 2 diabetes, atherosclerosis and cardiovascular diseases^36^. Adiponectin is a multifunctional adipokine, and is well studied for its roles in lipid metabolism and insulin resistance, and acts as an anti-inflammatory, anti-oxidative factor, and anti-fibrotic factor^30,37,38^. In animal studies, adiponectin was estimated to sensitize insulin in animals fed a high-fat diet^39,40^. Adiponectin promotes endothelial survival^41^, and ameliorated cardiac dysfunction and remodeling in ischemic hearts or limb in mouse models^28,29,42^. In adiponectin KO mice, cardiac injury was markedly exacerbated, while adiponectin supplementation exerts antiapoptotic and anti-inflammatory effects^43^. Although adiponectin is an adipocyte-specific endocrine molecule, adiponectin was also expressed in cardiomyocytes to protect from ischemia-reperfusion injury primarily via adiponectin receptor 1, AdipoR1 (refs. ^44,45^). In addition, we found that Retnla modulated the expressions of adiponectin and resistin. In cardiomyocytes, adiponectin was suppressed by Retnla treatment. On the contrary, both adiponectin and resistin were upregulated in Retnla-treated cardiac fibroblasts. Previous studies showed that adiponectin partially contributed to migration of fibroblasts^46^, and these results implicated the opposite roles of adiponectin and Retnla in terms of cardiac microenvironment.

To obtain translational potential, we examined the circulating adiponectin levels. Interestingly, adiponectin expression was upregulated in UBCs and heart tissues of Retnla KO mice, but there was a transient increase in Retnla KO mice at 2 days of MI without statistical significance. In humans, low plasma adiponectin levels are related to coronary artery disease, metabolic syndrome, and unfavorable cardiovascular risk profile^47,48^. High adiponectin levels are known to reduce MI risk and incident coronary heart disease events^49^. On the other hand, multiple lines of population-based studies showed conflicting associations of high circulating adiponectin levels with adverse cardiovascular outcomes, suggesting the “adiponectin paradox”. In spite of the abundant reports on the association of adiponectin levels with various populations, little is known about the clinical values of circulating adiponectin and localized expressions in specific organs in terms of cardiovascular disease. Although the effect of overexpressed Retnla is not overt so far, Retnla may participate in the feedback regulation of adiponectin mutually to maintain homeostasis within physiological levels.

We detected some interesting differences: the numbers of UBCs and cardiac macrophages were higher in Retnla KO mice, and notable increase of cell number led us to further investigate the involvement of UBCs in the disease progress post-MI. MMP2, survivin, Akt, and phosphorylated ERK substantially increased in UBCs from KO mice post-MI, and these results indicated that proliferation was better in KO-derived UBCs. To determine the proliferative activity of bone marrow, pH3, a marker for cell division, was used to quantify proliferating cells in bone marrow niche after MI. The numbers of pH3(+) cells were higher in the Retnla KO bone marrow than in the WT bone marrow at days 4 and 7 after MI. At 14 days, the proliferating cells were rarely observed in bone marrow from both WT and KO mice (data not shown). Of note, the mRNA level of adiponectin was higher in UBCs isolated from KO mice than in UBCs from WT mice. In addition, intrinsic angiogenic activity was compared by the tube forming assay with bone marrow-derived MSCs, and apparently showed high angiogenic capacity in stem cells isolated from Retnla KO bone marrow niche.

To investigate whether UBCs are determinant or not in the disease progress post-MI, an adoptive transfer study was performed. The cardiac function of recipient mice was apparently reflected that of donor mice, and these results implicated the UBCs were highly relevant to pathological progress after MI. Myeloid cells in the bone marrow are reported to be activated by MI. Myocardial injury resulted in monocyte recruitment in CCR2 signaling-dependent manner and CCR2(+) hematopoietic progenitor cells replicated robustly in bone marrow to release into circulation^21,50^. Furthermore, mobilization of hematopoietic and endothelial progenitor cells from bone marrow was stimulated by adiponectin^51,52^.

In the present study, Retnla KO mice showed cardioprotective features with the suppression of cardiomyocyte apoptosis, fibroblast proliferation, and systemic inflammation. Moreover, adiponectin-enriched UBCs might also contribute to improved repair capacity in Retnla KO mice.

Our study exemplifies the functional cross talk between cardiac microenvironment and bone marrow niche, and suggests that bone marrow niche can be harnessed to target cardiac injury and limit the pathological progress.

## Supplementary information

S Figure 1

S Figure 2

S Figure 3

S Figure 4

S Figure 5

S Figure 6

Supplementary Figure Legends

Supplementary Table 1

Supplementary Table 2
