# Supplementary figures and images for: The adipokine Retnla deficiency increases responsiveness to cardiac repair through adiponectin-rich bone marrow cells
Source: Cell Death Dis. 2021 Mar 22;12(4):307. doi: 10.1038/s41419-021-03593-z (PMC7985519; doi:10.1038/s41419-021-03593-z)

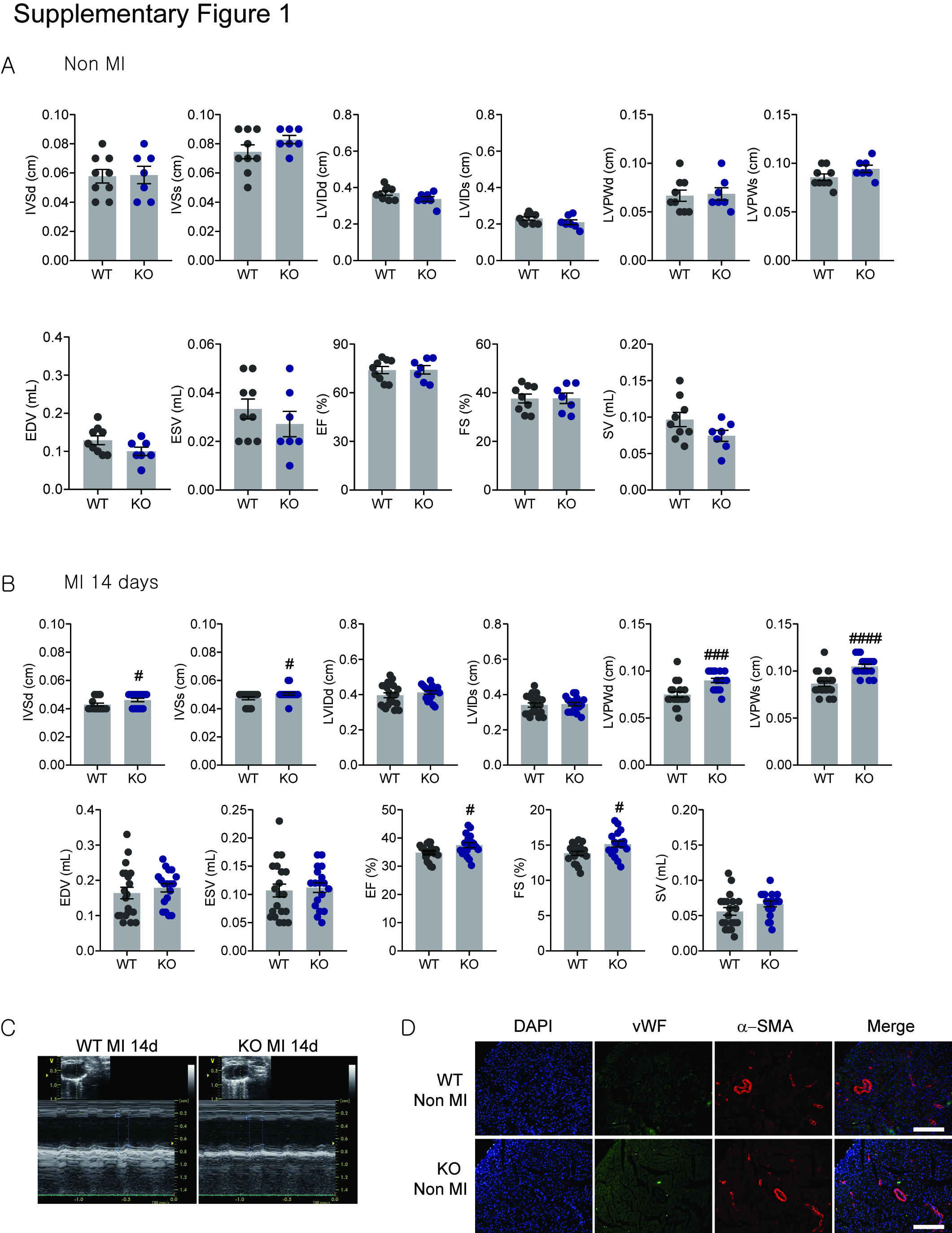

Supplement: Supplementary file 1 — S Figure 1 [file 41419_2021_3593_MOESM1_ESM.tif]

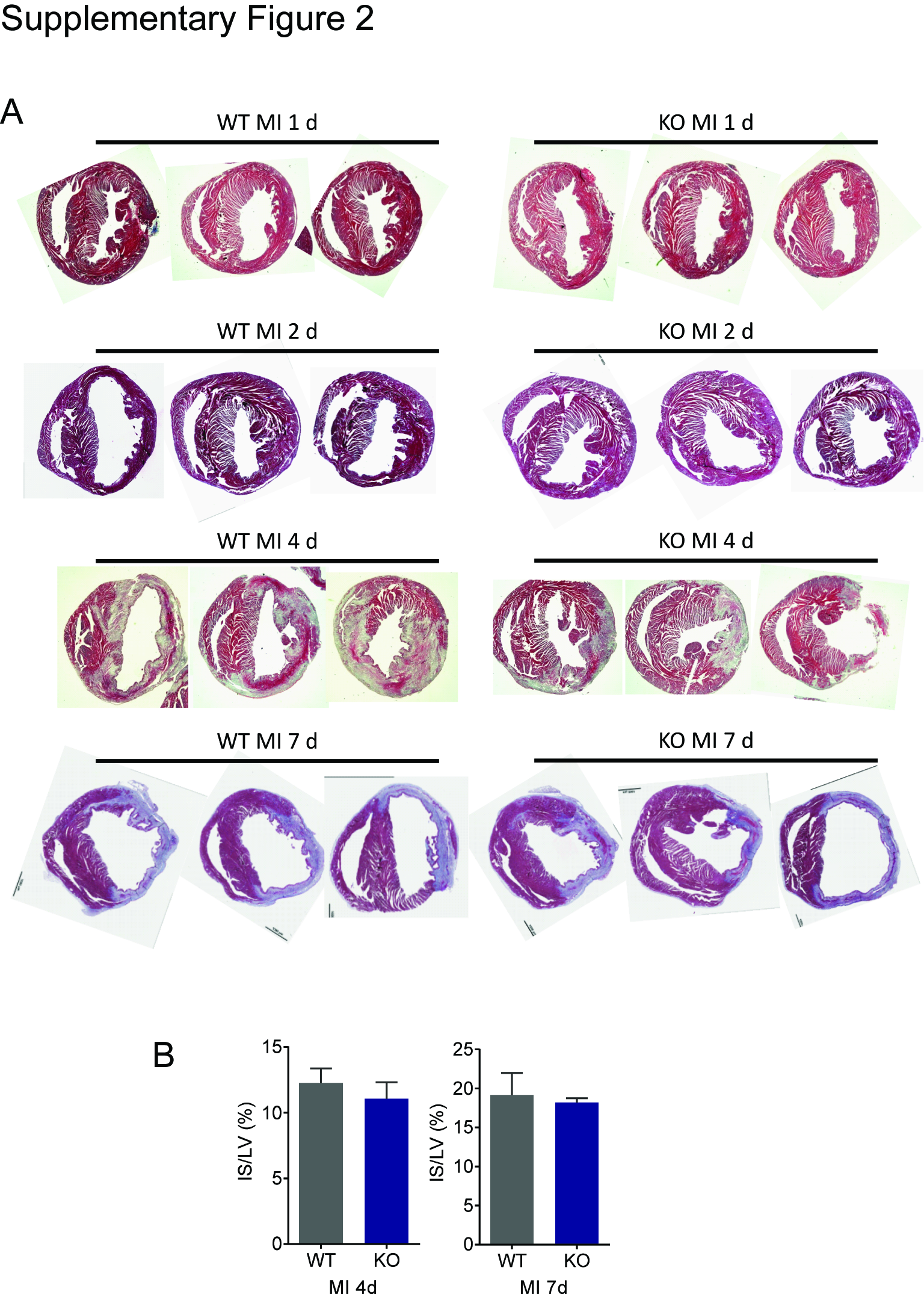

Supplement: Supplementary file 2 — S Figure 2 [file 41419_2021_3593_MOESM2_ESM.tif]

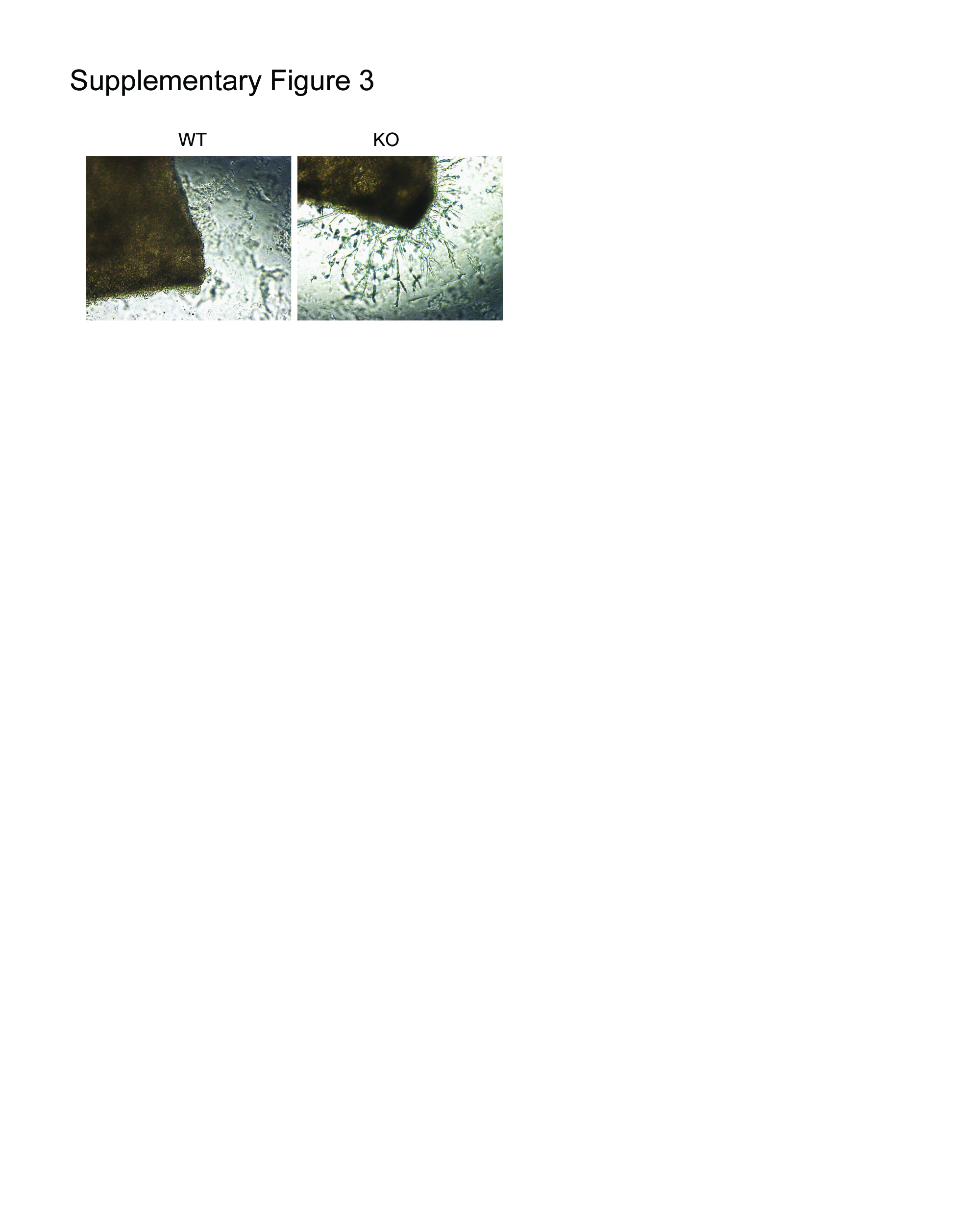

Supplement: Supplementary file 3 — S Figure 3 [file 41419_2021_3593_MOESM3_ESM.tif]

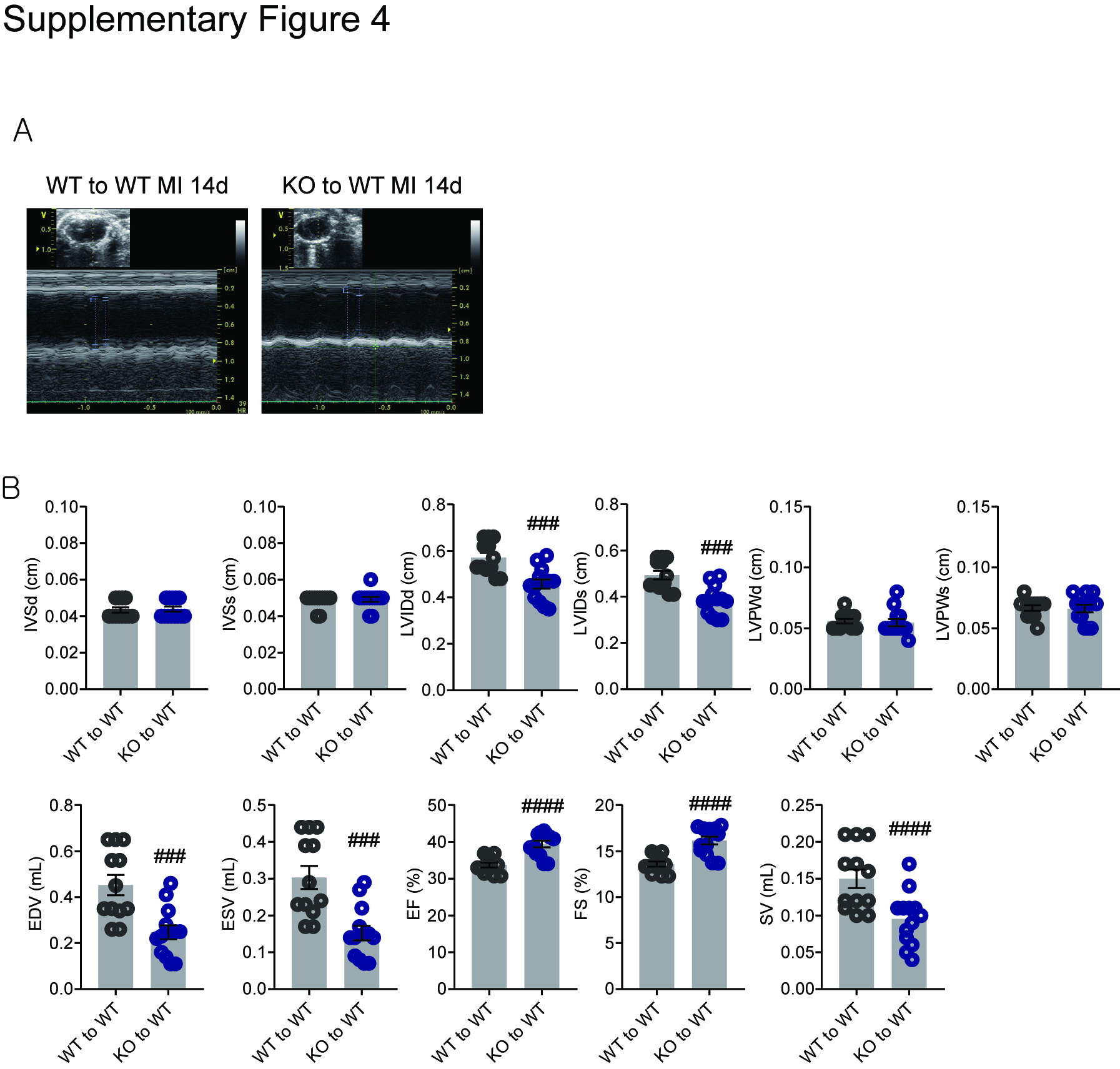

Supplement: Supplementary file 4 — S Figure 4 [file 41419_2021_3593_MOESM4_ESM.tif]

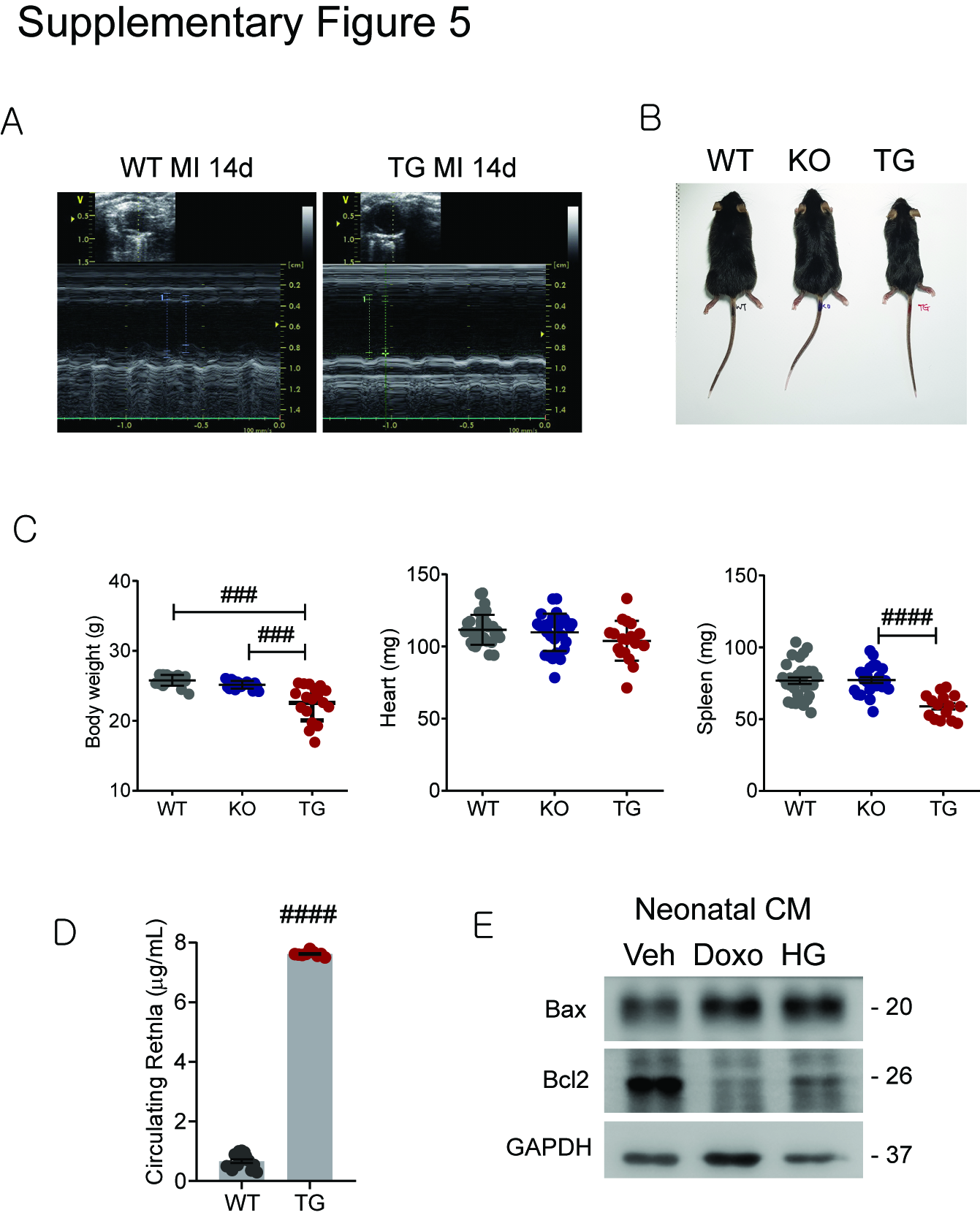

Supplement: Supplementary file 5 — S Figure 5 [file 41419_2021_3593_MOESM5_ESM.tif]

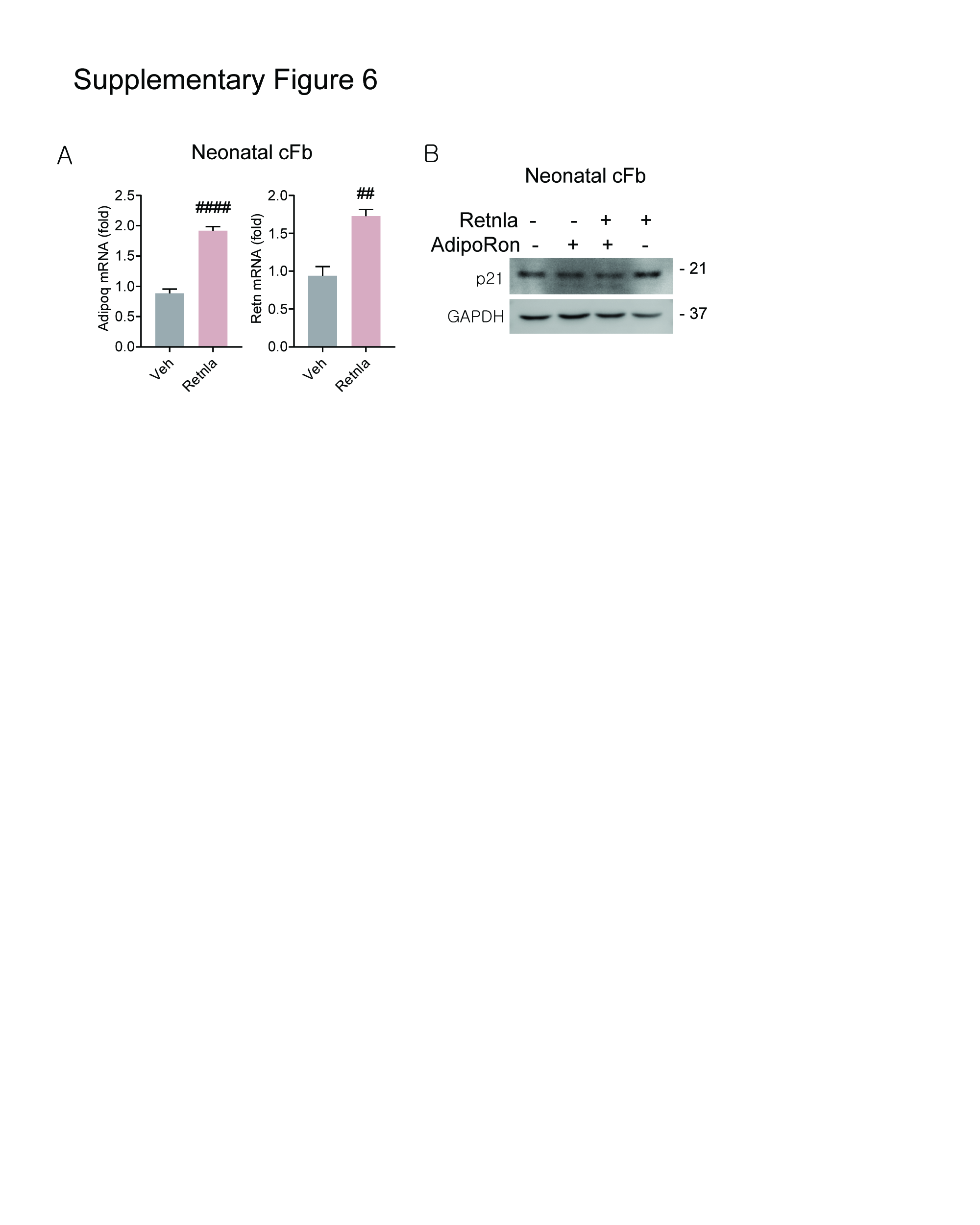

Supplement: Supplementary file 6 — S Figure 6 [file 41419_2021_3593_MOESM6_ESM.tif]
