# Supplementary material for: The adipokine Retnla deficiency increases responsiveness to cardiac repair through adiponectin-rich bone marrow cells
Source: Cell Death Dis. 2021 Mar 22;12(4):307. doi: 10.1038/s41419-021-03593-z (PMC7985519; doi:10.1038/s41419-021-03593-z)
Supplement: Supplementary file 7 — Supplementary Figure Legends [file 41419_2021_3593_MOESM7_ESM.docx]

**Supplementary Legends**

**Supplementary Figure 1.** Cardiac function measured by echocardiography at baseline (A, WT; n=9, KO; n=9) and 14 days (B, WT; n=9, KO; n=18) after MI surgery. (C) Representative echocardiograms were shown and left ventricular ejection fraction (LVEF) was determined by echocardiography in Retnla KO (n = 19) and WT littermates (n = 18) 14 days after MI. (D) Double immunohistochemistry for blood vessels were shown in WT mice and Retnla KO mice without MI. von Willebrand factor (vWF) is a small vessel marker and α-smooth muscle actin (α-SMA) is a large vessel marker. Scale bar=200μm. Data are represented as mean ± SEM. #P < 0.05, ##P < 0.01, ###P < 0.001 (by Student’s t test).

EF, ejection fraction; FS, fractional shortening; IVSd, intraventricular septal width in diastole; IVSs, intraventricular septal width in systole; LVIDd, left ventricular internal dimension in diastole; LVIDs, left ventricular internal dimension in systole; LVPWd, left ventricular posterior wall thickness in diastole; LVPWs, left ventricular posterior wall thickness in systole.

**Supplementary Figure 2. Left ventricular infarct and fibrosis.** (A) Masson trichrome staining on WT and Retnla KO mouse hearts at day 1, 2, 4, and 7 post-MI illustrates no significant differences.

(B) The areas of fibrotic scar were quantified. Data are represented as mean ± SEM. #P < 0.05, ##P < 0.01, ###P < 0.001 (by Student’s t test) WT; n=6, KO; n=6, WT MI 4d; n=5, KO MI 7d; n=4.

**Supplementary Figure 3. Angiogenesis *ex vivo*.** Angiogenesis *ex vivo* determined by aortic ring assay in WT mice and Retnla KO mice.

**Supplementary Figure 4. Cardiac function in an adoptive transfer study.** Unsorted bone marrow cells (UBCs) were isolated from WT mice (n=12) or KO mice (n=13), and then injected to infarcted heart of WT mice. (A) Representative echocardiograms were shown and (B) cardiac function was evaluated 14 days after MI.

EF, ejection fraction; FS, fractional shortening; IVSd, intraventricular septal width in diastole; IVSs, intraventricular septal width in systole; LVIDd, left ventricular internal dimension in diastole; LVIDs, left ventricular internal dimension in systole; LVPWd, left ventricular posterior wall thickness in diastole; LVPWs, left ventricular posterior wall thickness in systole. Data are represented as mean ± SEM. #P < 0.05, ##P < 0.01, ###P < 0.001 (by Student’s t test).

**Supplementary Figure 5. Phenotype of Retnla transgenic (TG) mouse.** (A) Representative echocardiograms were shown left ventricular ejection fraction (LVEF) was determined by echocardiography in Retnla transgenic mice (n = 20) and WT littermates (n = 13) 14 days after MI. (B, C) Retnla knockout (KO) mice were normal in appearance and body weight, but TG mice displayed lower body weight (WT n=18, KO n=18, TG n=18) and spleen weight (WT n=20, KO n=16, TG n=13). Heart weights were not different between the groups (WT n=34, KO n=28, TG n=18). (D) Circulating levels of Retnla was measured in the WT mice (n=15) and the TG mice (n=9). (E) Apoptosis of cardiomyocytes were induced by doxorubicin or high glucose treatment for 24 hours. Increase of pro-apoptotic Bax and decrease of anti-apoptotic Bcl-2 were clearly shown. Data are represented as mean ± SEM. #P < 0.05, ##P < 0.01, ###P < 0.001 (by Student’s t test or 1-way ANOVA with Bonferroni’s multiple comparisons test).

**Supplementary Figure 6. Effect of Retnla on cardiac cells.** (A) Effect of Retnla on mRNA expressions of adiponectin (Adipoq) and resistin (Retn) in neonatal cardiac fibroblasts. Cells were treated with veh (n=4) or Retnla protein (500 ng/mL, n=4) for 24 hours. (B) In Retnla-treated cardiac fibroblasts, downregulated p21 was restored by adiponectin receptor agonist AdipoRon. Cells were treated with Retnla protein (500 ng/mL) and AdipoRon (50 nM) for 24 hours. Data are represented as mean ± SEM. #P < 0.05, ##P < 0.01, ###P < 0.001 (by Student’s t test).
