# Supplementary material for: The adipokine Retnla deficiency increases responsiveness to cardiac repair through adiponectin-rich bone marrow cells
Source: Cell Death Dis. 2021 Mar 22;12(4):307. doi: 10.1038/s41419-021-03593-z (PMC7985519; doi:10.1038/s41419-021-03593-z)
Supplement: Supplementary file 8 — Supplementary Table 1 [file 41419_2021_3593_MOESM8_ESM.docx]

**Supplementary Table 1.** List of commercial antibodies

| Antibodies | Vendor | catalog No. |
| --- | --- | --- |
| Adiponectin | Abcam | ab22554 |
| Akt | Cell Signaling Technology | #9272 |
| Bax | Santa Cruz Biotechnology | sc-526 |
| Bcl2 | Cell Signaling Technology | #2876 |
| Cardiac troponin I | Santa Cruz Biotechnology | sc-15368 |
| CD206 | Abcam | ab64693 |
| Extracellular signal-regulated regulated kinase (ERK) | Cell Signaling Technology | #9102 |
| Phosphorylated ERK | Cell Signaling Technology | #9101 |
| GAPDH | Santa Cruz Biotechnology | sc-32233 |
| Ki67 | Abcam | ab15580 |
| Phosphorylated H3 histone | Abcam | ab32107 |
| iNOS | Cell Signaling Technology | #2982 |
| Matrix metalloproteinase (MMP) 2 | Santa Cruz Biotechnology | sc-13595 |
| α-smooth muscle actin | Sigma-Aldrich | A2547 |
| Survivin | Santa Cruz Biotechnology | sc-17779 |
| von Willebrand factor (vWF) | Sigma-Aldrich | F3520 |
| p21 | Abcam | ab2961 |
| p38 | Cell Signaling Technology | #9212 |
| Phosphorylated p38 | Cell Signaling Technology | #9211 |
| Mouse IgG, HRP-linked antibody | Cell Signaling Technology | #7076 |
| Rabbit IgG, HRP-linked antibody | Cell Signaling Technology | #7074 |
| Mouse IgG, Alexa Fluor 594 | ThermoFisher Scientific | A-11005 |
| Rabbit IgG, Alexa Fluor 594 | ThermoFisher Scientific | A-21207 |
| Mouse IgG, Alexa Fluor 488 | ThermoFisher Scientific | A-21200 |
| Rabbit IgG, Alexa Fluor 488 | ThermoFisher Scientific | A-11034 |
