# Supplementary material for: The adipokine Retnla deficiency increases responsiveness to cardiac repair through adiponectin-rich bone marrow cells
Source: Cell Death Dis. 2021 Mar 22;12(4):307. doi: 10.1038/s41419-021-03593-z (PMC7985519; doi:10.1038/s41419-021-03593-z)
Supplement: Supplementary file 9 — Supplementary Table 2 [file 41419_2021_3593_MOESM9_ESM.docx]

**Supplemental Table 2.** Primers for real time-PCR

| Gene | Primer |
| --- | --- |
| Mouse *Adipoq* | Forward : 5’-GGCAGGAAAGGAGAACCTGG-3’  Reverse: 5’-AGCCTTGTCCTTCTTGAAGAG-3’ |
| Mouse *Bad* | Forward : 5’-CAGCCACCAACAGTCATCAT-3’  Reverse: 5’-CTCAAACTCATCGCTCATCCTT-3’ |
| Mouse *Bax* | Forward : 5’-TTTGCTACAGGGTTTCATCCA-3’  Reverse: 5’-CAAAGTAGAAGAGGGCAACCA-3’ |
| Mouse *Cav1* | Forward : 5’-ATTGCAGAACCAGAAGGGACAC-3’  Reverse: 5’-CCATTGGGATGCCGAAGAT-3’ |
| Mouse *Cdkn1a* | Forward : 5’-CACAGCTCAGTGGACTGGAA-3’  Reverse: 5’-CCACCACCACACACCATAGA-3’ |
| Mouse *Col4a5* | Forward : 5’-ACTGCCAGGACCAAAGGGTAATCA-3’  Reverse: 5’-TGGTTGGCCAGTCTCTCCATCTTT-3’ |
| Mouse *Col5a3* | Forward : 5’-ATTGGATTTCCTGGACCCTTGGGA-3’  Reverse: 5’-TCTATGCCTGGCAGACCTTGTTCA-3’ |
| Mouse *Faslg* | Forward : 5’-TGCAGCAGCCCATGAATTA-3’  Reverse: 5’-CCTAATCCCATTCCAACCAGAG-3’ |
| Mouse *Gapdh* | Forward : 5’-GGGTGTGAACCACGAGAAATA-3’  Reverse: 5’-GTCATGAGCCCTTCCACAAT-3’ |
| Mouse *Il6* | Forward : 5’-GAGGATACCACTCCCAACAGACC -3’  Reverse: 5’-AAGTGCATCATCGTTGTTCATACA - 3’ |
| Mouse *Il10* | Forward : 5’-TTGAATTCCCTGGGTGAGAAG-3’  Reverse: 5’-TCCACTGCCTTGCTCTTATTT-’3’ |
| Mouse *Retn* | Forward : 5’-ATGAAGCCATCGACAAGAAGAT-3’  Reverse: 5’-GTCCAGTCTATCCTTGCACAC-3’ |
| Mouse *Retnla* | Forward : 5’-CAGTTGCAAGTATCTCCACTCT-3’  Reverse: 5’-AGACTACAACTTGTTCCCTTCTC-3’ |
| Mouse *Tgfb1* | Forward : 5’-CTGAACCAAGGAGACGGAATAC-3’  Reverse: 5’-CTCTGTGGAGCTGAAGCAATAG-3’ |
| Mouse *Tlr2* | Forward : 5’-TCTGAGAATGATGTGGGCGT-3’  Reverse: 5’-GGGCCACTCCAGGTATGTCT-3’ |
| Mouse *Tnf* | Forward : 5’-CGTCAGCCGATTTGCTATCT-3'  Reverse: 5’-AGCAATGACTCCAAAGTAGACC-3' |
| Rat *Adipoq* | Forward : 5’-CCCCTGGCAGGAAAGGA-3’  Reverse: 5’-TCCAGCCCTACGCTGAATG-3’ |
| Rat *Ccnd2* | Forward : 5’-GCTCTGTGTGCTACCGACTT-3’  Reverse: 5’-GGTCCGGATCTTCCACAGAC-3’ |
| Rat *Ccne1* | Forward : 5’-GATCCAGAAAAAGGAAGCCAAA-3’  Reverse: 5’-TGTCAATCTTGGCAATTTCTTCA-3’ |
| Rat *Cdkn1a* | Forward : 5’-CCAAACACCCCTGTTTCTGT-3’  Reverse: 5’-TTTACAATCCGAGCGGAGAC-3’ |
| Rat *Cdkn3* | Forward : 5’-TTCCCCGGATACCACACAAG-3’  Reverse: 5’-ACAGCAGAGGGGGAGGTTAG-3’ |
| Rat *Gapdh* | Forward : 5’-GGCCAAGGTCATCCATGA-3’  Reverse: 5’-TCAGTGAGCCCAGGATG-3’ |
| Rat *Nppb* | Forward : 5’-ATGGATCTCCTGAAGGTGCTG -3’  Reverse: 5’-GTGCTGCCTTGAGACCGAA-3’ |
| Rat *Retn* | Forward : 5’-ACTTCAGCTCCCTACTG-3’  Reverse: 5’-GTCTATGCTTCCGCACT-3’ |
| Rat *Tnfrsf12a* | Forward : 5’-ATGGACTGCGCTTCTTGTC-3’  Reverse: 5’-ATGGACTGCGCTTCTTGTC-3’ |
